# (*E*)-4-[(4-Bromo­benzyl­idene)amino]phenol

**DOI:** 10.1107/S160053680905538X

**Published:** 2010-01-09

**Authors:** Jasmine P. Vennila, D. John Thiruvadigal, Helen P. Kavitha, B. Gunasekaran, V. Manivannan

**Affiliations:** a Department of Physics, Panimalar Institute of Technology, Chennai 602 103, India; b Department of Physics, SRM University, Kattankulathur Campus, Chennai, India; c Department of Chemistry, SRM University, Ramapuram Campus, Chennai 600 089, India; d Department of Physics, AMET University, Kanathur, Chennai 603 112, India; e Department of Research and Development, PRIST University, Vallam, Thanjavur 613 403, Tamil Nadu, India

## Abstract

In the title compound, C_13_H_10_BrNO, the dihedral angle between the benzene rings is 35.20 (8)°. In the crystal, mol­ecules are linked by O—H⋯N hydrogen bonds, forming a zigzag chain along the *a* axis. A weak C—H⋯π inter­action is observed between the chains.

## Related literature

For the biological activity of benzyl­idene derivatives, see: El Masry *et al.* (2000 ▶); Fegade *et al.* (2009 ▶); Foroumadi *et al.* (2007 ▶); Hodnett & Dunn (1970 ▶); Hu & Zhou (2004 ▶); Jada *et al.* (2008 ▶); Samadhiya & Halve (2001 ▶); Singh & Dash (1988 ▶). For related structures, see: Cui *et al.* (2009 ▶); Sun *et al.* (2009 ▶). For bond-length data, see: Allen *et al.* (1987 ▶).
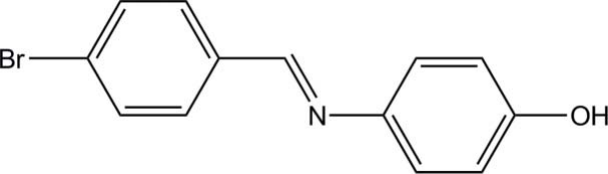


## Experimental

### 

#### Crystal data

C_13_H_10_BrNO
*M* *_r_* = 276.13
Orthorhombic, 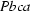

*a* = 12.7035 (4) Å
*b* = 10.3897 (3) Å
*c* = 17.0899 (6) Å
*V* = 2255.62 (12) Å^3^
*Z* = 8
Mo *K*α radiation
μ = 3.62 mm^−1^
*T* = 295 K
0.20 × 0.16 × 0.15 mm

#### Data collection

Bruker Kappa APEXII CCD diffractometer
Absorption correction: multi-scan (*SADABS*; Sheldrick, 1996 ▶) *T*_min_ = 0.503, *T*_max_ = 0.581
13273 measured reflections
2670 independent reflections
1710 reflections with *I* > 2σ(*I*)
*R*_int_ = 0.043

#### Refinement

*R*[*F* ^2^ > 2σ(*F* ^2^)] = 0.035
*wR*(*F* ^2^) = 0.086
*S* = 1.00
2670 reflections
146 parameters
H-atom parameters constrained
Δρ_max_ = 0.49 e Å^−3^
Δρ_min_ = −0.40 e Å^−3^

### 

Data collection: *APEX2* (Bruker, 2004 ▶); cell refinement: *SAINT* (Bruker, 2004 ▶); data reduction: *SAINT*; program(s) used to solve structure: *SHELXS97* (Sheldrick, 2008 ▶); program(s) used to refine structure: *SHELXL97* (Sheldrick, 2008 ▶); molecular graphics: *PLATON* (Spek, 2009 ▶); software used to prepare material for publication: *SHELXL97*.

## Supplementary Material

Crystal structure: contains datablocks global, I. DOI: 10.1107/S160053680905538X/is2508sup1.cif

Structure factors: contains datablocks I. DOI: 10.1107/S160053680905538X/is2508Isup2.hkl

Additional supplementary materials:  crystallographic information; 3D view; checkCIF report

## Figures and Tables

**Table 1:** Hydrogen-bond geometry (Å, °) *Cg*1 is the centroid of the C8–C13 ring.

*D*—H⋯*A*	*D*—H	H⋯*A*	*D*⋯*A*	*D*—H⋯*A*
O1—H1⋯N1^i^	0.82	2.05	2.848 (3)	164
C5—H5⋯*Cg*1^ii^	0.93	2.89	3.374 (3)	114

Symmetry codes: (i) 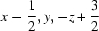; (ii) 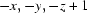.

**Fig. 1.**
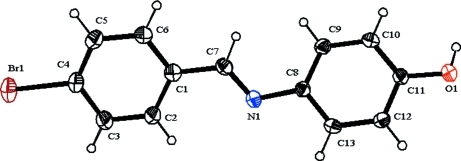
The molecular structure of the title compound, with atom labels and 30% probability displacement ellipsoids for non-H atoms.

## supplementary crystallographic information

### Comment

Benzylidene derivatives exhibit antitumor (Hu & Zhou 2004) and antioxidant (Foroumadi *et al.*, 2007) activities. Some *N*-benzylidene aniline derivatives show biological activities sucs as antibacterial (El Masry *et al.*, 2000), antifungal (Singh & Dash, 1988), anticancer (Hodnett & Dunn, 1970) and herbicidal (Samadhiya & Halve, 2001). In addition, benzylidene derivatives of andrographolide are potential anticancer agents (Jada *et al.*, 2008) and some of the benzylidene derivatives are acting as selective cyclooxygenase-2-inhibitors (Fegade *et al.*, 2009).

The geometric parameters of the title compound (Fig. 1) agree well with reported similar structures (Cui *et al.*, 2009; Sun *et al.*, 2009). The dihedral angle between the benzene rings is 35.20 (8)°. The C—Br bond distance is 1.894 (2) Å, which is comparable to the literature value of 1.883 (15) Å (Allen *et al.*, 1987). The crystal packing is stabilized by an O—H···N hydrogen bond and a weak C—H···π interaction (Table 1).

### Experimental

A mixture of 4-bromobenzaldehyde (5 mmol), 4-aminophenol (5 mmol) and ethanol (40 ml) was refluxed for 2 h. It was then allowed to cool and filtered. Recrystallization of the crude product from ethanol yielded brown colored crystals.

### Refinement

H atoms were positioned geometrically and refined using riding model, with O—H = 0.82 Å and *U*_iso_(H) = 1.2*U*_eq_(O), and C—H = 0.93 Å and *U*_iso_(H) = 1.2*U*_eq_(C).

### Crystal data

**Table:**

C_13_H_10_BrNO	*F*(000) = 1104
*M**_r_* = 276.13	*D*_x_ = 1.626 Mg m^−^^3^
Orthorhombic, *P**b**c**a*	Mo *K*α radiation, λ = 0.71073 Å
Hall symbol: -P 2ac 2ab	Cell parameters from 2371 reflections
*a* = 12.7035 (4) Å	θ = 2.4–23.7°
*b* = 10.3897 (3) Å	µ = 3.62 mm^−^^1^
*c* = 17.0899 (6) Å	*T* = 295 K
*V* = 2255.62 (12) Å^3^	Block, brown
*Z* = 8	0.20 × 0.16 × 0.15 mm

### Data collection

**Table:**

Bruker Kappa APEXII CCD diffractometer	2670 independent reflections
Radiation source: fine-focus sealed tube	1710 reflections with *I* > 2σ(*I*)
graphite	*R*_int_ = 0.043
ω and φ scans	θ_max_ = 27.8°, θ_min_ = 2.4°
Absorption correction: multi-scan (*SADABS*; Sheldrick, 1996)	*h* = −12→16
*T*_min_ = 0.503, *T*_max_ = 0.581	*k* = −12→13
13273 measured reflections	*l* = −22→22

### Refinement

**Table:**

Refinement on *F*^2^	Primary atom site location: structure-invariant direct methods
Least-squares matrix: full	Secondary atom site location: difference Fourier map
*R*[*F*^2^ > 2σ(*F*^2^)] = 0.035	Hydrogen site location: inferred from neighbouring sites
*wR*(*F*^2^) = 0.086	H-atom parameters constrained
*S* = 1.00	*w* = 1/[σ^2^(*F*_o_^2^) + (0.0358*P*)^2^ + 0.8359*P*] where *P* = (*F*_o_^2^ + 2*F*_c_^2^)/3
2670 reflections	(Δ/σ)_max_ = 0.001
146 parameters	Δρ_max_ = 0.49 e Å^−^^3^
0 restraints	Δρ_min_ = −0.39 e Å^−^^3^

### Special details

**Table:**

Geometry. All e.s.d.'s (except the e.s.d. in the dihedral angle between two l.s. planes) are estimated using the full covariance matrix. The cell e.s.d.'s are taken into account individually in the estimation of e.s.d.'s in distances, angles and torsion angles; correlations between e.s.d.'s in cell parameters are only used when they are defined by crystal symmetry. An approximate (isotropic) treatment of cell e.s.d.'s is used for estimating e.s.d.'s involving l.s. planes.

### Fractional atomic coordinates and isotropic or equivalent isotropic displacement parameters (Å^2^)

**Table:**

	*x*	*y*	*z*	*U*_iso_*/*U*_eq_	
C1	0.16600 (18)	0.0270 (2)	0.54592 (14)	0.0371 (6)	
C2	0.2620 (2)	−0.0367 (3)	0.54384 (16)	0.0463 (7)	
H2	0.2727	−0.1086	0.5753	0.056*	
C3	0.34124 (19)	0.0056 (3)	0.49578 (18)	0.0520 (7)	
H3	0.4054	−0.0374	0.4944	0.062*	
C4	0.32501 (19)	0.1120 (3)	0.44968 (15)	0.0422 (6)	
C5	0.2319 (2)	0.1769 (3)	0.45068 (16)	0.0463 (7)	
H5	0.2220	0.2490	0.4192	0.056*	
C6	0.1528 (2)	0.1340 (3)	0.49907 (16)	0.0449 (7)	
H6	0.0891	0.1780	0.5002	0.054*	
C7	0.07751 (18)	−0.0183 (3)	0.59347 (15)	0.0383 (6)	
H7	0.0158	0.0298	0.5927	0.046*	
C8	−0.01245 (17)	−0.1551 (2)	0.67752 (14)	0.0325 (5)	
C9	−0.08369 (16)	−0.0690 (2)	0.70991 (15)	0.0365 (6)	
H9	−0.0736	0.0189	0.7031	0.044*	
C10	−0.16897 (17)	−0.1120 (2)	0.75198 (14)	0.0368 (6)	
H10	−0.2155	−0.0532	0.7740	0.044*	
C11	−0.18585 (16)	−0.2426 (2)	0.76165 (15)	0.0360 (6)	
C12	−0.11568 (19)	−0.3294 (2)	0.72949 (16)	0.0412 (6)	
H12	−0.1267	−0.4174	0.7355	0.049*	
C13	−0.02907 (18)	−0.2855 (2)	0.68836 (15)	0.0388 (6)	
H13	0.0186	−0.3443	0.6677	0.047*	
N1	0.07946 (14)	−0.1183 (2)	0.63553 (12)	0.0367 (5)	
O1	−0.26777 (13)	−0.29148 (18)	0.80295 (12)	0.0497 (5)	
H1	−0.3015	−0.2324	0.8226	0.075*	
Br1	0.43298 (2)	0.16639 (3)	0.38080 (2)	0.06483 (15)	

### Atomic displacement parameters (Å^2^)

**Table:**

	*U*^11^	*U*^22^	*U*^33^	*U*^12^	*U*^13^	*U*^23^
C1	0.0383 (13)	0.0383 (15)	0.0348 (14)	−0.0034 (11)	−0.0019 (11)	−0.0038 (12)
C2	0.0458 (14)	0.0461 (16)	0.0471 (16)	0.0036 (12)	0.0056 (13)	0.0117 (14)
C3	0.0386 (14)	0.0573 (19)	0.0599 (19)	0.0045 (13)	0.0086 (14)	0.0115 (15)
C4	0.0427 (14)	0.0468 (16)	0.0371 (15)	−0.0113 (12)	0.0046 (12)	0.0009 (13)
C5	0.0549 (16)	0.0432 (16)	0.0407 (16)	−0.0024 (13)	0.0003 (13)	0.0081 (13)
C6	0.0406 (14)	0.0464 (17)	0.0476 (17)	0.0047 (11)	0.0009 (13)	0.0066 (13)
C7	0.0322 (13)	0.0420 (16)	0.0406 (14)	0.0007 (10)	−0.0006 (11)	−0.0023 (13)
C8	0.0268 (11)	0.0357 (14)	0.0349 (13)	−0.0010 (10)	−0.0028 (10)	0.0021 (11)
C9	0.0337 (12)	0.0305 (13)	0.0452 (16)	−0.0010 (10)	−0.0018 (11)	0.0006 (12)
C10	0.0332 (13)	0.0360 (15)	0.0411 (15)	0.0066 (10)	−0.0012 (11)	−0.0013 (12)
C11	0.0291 (12)	0.0387 (16)	0.0403 (15)	0.0004 (10)	0.0010 (11)	0.0041 (11)
C12	0.0361 (12)	0.0308 (14)	0.0566 (18)	0.0013 (11)	0.0019 (12)	0.0056 (13)
C13	0.0319 (12)	0.0364 (15)	0.0480 (16)	0.0076 (11)	0.0022 (11)	0.0021 (13)
N1	0.0314 (10)	0.0404 (12)	0.0384 (13)	−0.0021 (8)	0.0016 (9)	0.0000 (10)
O1	0.0371 (10)	0.0431 (11)	0.0688 (14)	0.0020 (8)	0.0169 (9)	0.0058 (10)
Br1	0.0582 (2)	0.0736 (3)	0.0627 (2)	−0.01461 (15)	0.01935 (16)	0.01033 (18)

### Geometric parameters (Å, °)

**Table:**

C1—C6	1.380 (4)	C8—C13	1.383 (3)
C1—C2	1.388 (3)	C8—C9	1.388 (3)
C1—C7	1.465 (3)	C8—N1	1.423 (3)
C2—C3	1.371 (4)	C9—C10	1.375 (3)
C2—H2	0.9300	C9—H9	0.9300
C3—C4	1.373 (4)	C10—C11	1.383 (4)
C3—H3	0.9300	C10—H10	0.9300
C4—C5	1.362 (4)	C11—O1	1.356 (3)
C4—Br1	1.894 (2)	C11—C12	1.382 (3)
C5—C6	1.376 (4)	C12—C13	1.383 (3)
C5—H5	0.9300	C12—H12	0.9300
C6—H6	0.9300	C13—H13	0.9300
C7—N1	1.264 (3)	O1—H1	0.8200
C7—H7	0.9300		
			
C6—C1—C2	118.4 (2)	C13—C8—C9	118.6 (2)
C6—C1—C7	119.2 (2)	C13—C8—N1	117.1 (2)
C2—C1—C7	122.4 (2)	C9—C8—N1	124.3 (2)
C3—C2—C1	120.5 (3)	C10—C9—C8	120.9 (2)
C3—C2—H2	119.8	C10—C9—H9	119.6
C1—C2—H2	119.8	C8—C9—H9	119.6
C2—C3—C4	119.4 (2)	C9—C10—C11	120.2 (2)
C2—C3—H3	120.3	C9—C10—H10	119.9
C4—C3—H3	120.3	C11—C10—H10	119.9
C5—C4—C3	121.5 (2)	O1—C11—C12	117.2 (2)
C5—C4—Br1	119.3 (2)	O1—C11—C10	123.3 (2)
C3—C4—Br1	119.2 (2)	C12—C11—C10	119.5 (2)
C4—C5—C6	118.7 (3)	C11—C12—C13	120.0 (2)
C4—C5—H5	120.6	C11—C12—H12	120.0
C6—C5—H5	120.6	C13—C12—H12	120.0
C5—C6—C1	121.4 (2)	C12—C13—C8	120.8 (2)
C5—C6—H6	119.3	C12—C13—H13	119.6
C1—C6—H6	119.3	C8—C13—H13	119.6
N1—C7—C1	124.4 (2)	C7—N1—C8	119.4 (2)
N1—C7—H7	117.8	C11—O1—H1	109.5
C1—C7—H7	117.8		
			
C6—C1—C2—C3	−0.5 (4)	N1—C8—C9—C10	−177.8 (2)
C7—C1—C2—C3	177.0 (3)	C8—C9—C10—C11	−1.0 (4)
C1—C2—C3—C4	0.1 (5)	C9—C10—C11—O1	179.4 (2)
C2—C3—C4—C5	0.2 (5)	C9—C10—C11—C12	0.8 (4)
C2—C3—C4—Br1	−177.9 (2)	O1—C11—C12—C13	−178.4 (2)
C3—C4—C5—C6	−0.1 (4)	C10—C11—C12—C13	0.3 (4)
Br1—C4—C5—C6	177.9 (2)	C11—C12—C13—C8	−1.2 (4)
C4—C5—C6—C1	−0.2 (4)	C9—C8—C13—C12	1.0 (4)
C2—C1—C6—C5	0.5 (4)	N1—C8—C13—C12	179.1 (2)
C7—C1—C6—C5	−177.0 (3)	C1—C7—N1—C8	−178.2 (2)
C6—C1—C7—N1	176.6 (3)	C13—C8—N1—C7	147.6 (3)
C2—C1—C7—N1	−0.9 (4)	C9—C8—N1—C7	−34.5 (4)
C13—C8—C9—C10	0.1 (4)		

### Hydrogen-bond geometry (Å, °)

**Table:**

Cg1 is the centroid of the C8–C13 ring.

**Table:**

*D*—H···*A*	*D*—H	H···*A*	*D*···*A*	*D*—H···*A*
O1—H1···N1^i^	0.82	2.05	2.848 (3)	164
C5—H5···Cg1^ii^	0.93	2.89	3.374 (3)	114

Symmetry codes: (i) *x*−1/2, *y*, −*z*+3/2; (ii) −*x*, −*y*, −*z*+1.
